# Editor's choice: Sexual reproductive health, child issues, NCDs, surgery, infections and health systems - a salad of sorts

**DOI:** 10.4314/ahs.v23i3.1

**Published:** 2023-09

**Authors:** James K Tumwine

This September 2023 issue of African Health Sciences is special in that it brings you fairly sophisticated papers ranging from those in sexual reproductive and child health, non-communicable diseases, infections, surgery, mental health and others.

The sexual reproductive health papers touch on a number of issues. Hence we have one on the efficacy of intra umbilical oxytocin^1^, pain relief in manual vacuum extraction^2^, emergency caesarean section^3^, and postnatal hypertension^4^. This is followed by prenatal papers^5^-^6^. Others highlight teenage pregnancy^7^, contraception and fertility issues^8^-^9^. Health challenges of menopause, and pre-menopause hold a special place^10^-^11^ as are social behavior and violence against women and the girl child^12^-^14^.

Child Health, on the other hand, is dominated by neonatal issues such as birth asphyxia^15^-^16^, low birth weight^17^, and acute respiratory distress syndrome^18^. Issues affecting the child include under five mortality^19^, nutrition^20^, vaccination^21^, use of mHealth^22^, iron deficiency^23^, sickle cell anemia^24^, and domestic accidents^25^. We have a large collection on cancers^26^-^34^, including breast, ovarian and cervical, and pulmonary lesions, while diabetes mellitus papers^35^-^37^ occupy the next space.

As usual, infections^38^-^56^ are ubiquitous and don't seem to be going away. They include papers on tuberculosis^38^-^42^, while most^43^-^49^ are dominated by Covid-19. The rest of this section consists of a heterogenous group of other infectious diseases^50^-^56^. We have a special section for those interested in Surgery and anesthesia^57^-^70^. The rest of the papers are in the realm of internal medicine^71^-^79^, mental health^80^-^81^ and health systems^82^-^87^.

Finally, it only remains for me to thank all our partners for the friendship and confidence they have placed in us as we strive to push African scientific publishing to a higher level. Thank you very much indeed. Happy reading!
